# The creative city approach: origins, construction and prospects in a scenario of transition

**DOI:** 10.1186/s40410-022-00178-x

**Published:** 2022-10-07

**Authors:** Chema Segovia, Julie Hervé

**Affiliations:** 1 Máster Universitario en Ciudad y Urbanismo, Universitat Oberta de Catalunya, Barcelona, Spain; 2 Facultat de Ciències Socials, Universitat de València, Valencia, Spain; 3 Eurocities, Brussels, Belgium

**Keywords:** Creative city, Urban policy, Cultural policy, Sustainability, Transition

## Abstract

The change of the century saw the emergence of a series of discourses that conceptualised different aspects related with culture as key elements in the future of urban realities. The fact that these notions have become encompassed within the celebrated label of “the creative city” leads us to think that they form a self-evident model, fully assimilated and of general value. However, the review of the process through which a reasonably cohesive and accepted framework was constructed unveils the complex nature of the creative city. This article introduces the idea of the creative city as an “approach”, in the sense of an epistemological and methodological focus that is distinguishable, yet neither rigid nor closed. An understanding of this type is useful for assessing the validity and the imbalances of the creative city in the midst of an epoch of problematic transition, in which culture and the city are alternatively defined as spaces of conflict or spaces of hope.

## Introduction

Between the early 1990 s and the first years of the 21st century, local and regional development theories saw the emergence of a series of discourses that conceptualised different aspects related with culture as key elements in the future of urban realities (Scott 1997; Landry 2000; Evans 2001; Florida 2002). In a slightly problematic way, due to the loss of nuance, although acceptably operational, these notions were encompassed within the label of “the creative city”.

While the successful reception and enthusiastic dissemination of these discourses acquired hues of a policymaking fad, at present we can affirm that their influence has become long-lasting. Nowadays, intense attention continues to be paid to the importance of the cultural dimension of urban environments (Pratt 2014; Rausell-Köster 2015; Rodrigues and Franco 2018), numerous cities and regions design policies that seek to act on that sphere (Culture for Cities and Regions 2015; Kagan et al. 2018) and the main international organisations recognise the central importance of culture, creativity and innovation in urban governance and city development (OECD 2018; UNCTAD 2019; UNESCO & World Bank 2021).

Paradoxically, in spite of this broad acceptance, the idea of the creative city is far from becoming enshrined within clearly defined limits. A point important to underline is that at no time has it been nor is it likely to become so. The ideas that hold up the creative city framework possess a polyhedral and even contradictory nature. In addition, the shape of this polyhedron has shifted over time, adding new vertices and corners depending on different changes of comprehension (Evans 2017). The recognition of the creative city as a field for research and action must acknowledge its tangled, oscillating and slightly vague status. This particularity makes it necessary to regularly adjust its conceptual baselines and review its evolution, with these being two exercises that define a specific area within the studies of creative cities (Bianchini 2004; Chatterton 2010; Landry 2012; Grodach and Silver 2013; Pratt and Hutton 2013; Markusen 2014; D’Ovido 2016; Banks and O’Connor 2017).

The contribution that this article hopes to make is centred on this area and is supported by a key argument: faced with the extended idea that considers the creative city to be defined as a self-evident model, fully assimilated and of general value (Van Damme and De Munck 2018), the review of its genealogy and its transnational validation reveals that it is more appropriate to talk in terms of “approach”, in the sense of a distinguishable epistemological and methodological focus, but one that is neither rigid nor closed.

Furthermore, paying attention to the process through which the creative city framework was assembled places its emergence within a specific geographical context and epoch, where many different factors coincide, having a profound influence on that framework’s configuration. Although the evolution that followed the articulation of a baseline notion is read in terms of enrichment and sophistication, it can also be argued that to a good extent, the core of the creative city framework, the point where its main bents and dilemmas lie, still remains the same as that which was constructed with the turn of the century.

The introduction of an idea of the creative city as an approach which, despite having evolved over time is deeply rooted within a specific context, serves as a bridge to the argument where this paper redoubles its relevance, which means using historical reflection to critically question and revise the usefulness of the creative city nowadays. Relating the period in which the creative city emerged and the moment that we are living is especially relevant from two perspectives. On the one hand, as regards difference, we understand that the permanent crisis that began in 2008 and which has been reaffirmed by the Covid-19 pandemic affects the validity of a part of the founding statements of the creative city. And on the other, speaking in terms of similitude, we observe that the cycle in which the creative city framework was proposed and validated has a direct relationship with the current time, given that they both share the fact that the understanding of the ideas of city and culture are objects of discussion and reformulation.

This paper thus proposes an exercise divided into two parts. We will begin by describing the process through which the creative city policy proposal emerged and was integrated into the transnational agendas for the future as a distinctive but malleable framework. We will do so by using a broad perspective, particularly focusing on the understandings, debates and inertias that were influential in the articulation of such a framework. Once an idea of the creative city as an approach is formulated, we will re-situate it in the current transition scenario. We will list the cultural challenges large western cities -those that played an active role in the introduction of the notion of urban creativity- face nowadays, and we will identify the positive trends of change with which the creative city, conceived as an approach, could establish links of mutual renovation and reinforcement. The global objective of this paper is to infuse some open-ended historical reflexivity in order to appraise the current validity of the creative city. To conclude, we will propose a series of key issues for a new insight.

## Constructing the creative city approach

The articulation of the creative city as a relatively cohesive framework is inscribed in the span of time which goes from the 1970 s to the first decade of the present century. As we will see, the United Kingdom played a key role in this process, it being necessary to also consider the weight that the European region exercised when adapting and validating a policy proposal that has obtained a global dimension.

Although our description is mainly linear, it is important to stress that the construction of the creative city approach is better understood as an organic process, carried out on different fronts, based on the imperfect combination of disparate ideas, characterised by multiple ramifications and overlaps. The exposition below aims to account for this.

### The basis of the creative city vision: cities and culture in a post-industrial world

The first formulation of the creative city was influenced by a series of assumptions which, in an agitated and not particularly placid manner, took shape during the process of economic and political restructuring fed by the acceleration of globalisation in the last decades of the 20th century.

From the economic perspective, after the decline of the Fordist regime, the announcement of the arrival of a post-industrial world (Bell 1976) caused a strong change of patterns in the manner of observing reality and imagining the future. Although there were voices that warned of the risks of a too literal and deterministic reading of those descriptions (Cohen and Zysman 1987), the narratives from that period outlined an idea of development built upon “the end of industry”. From that moment onwards, the western states sought to change their productive models towards a globally competitive service economy.

As for the political aspect, the post-fordism roll-out coincided with the growing questioning of the Welfare State and the subsequent expansion of neoliberalism (Kus 2006). The phase between the 1970 s and the 1980 s showed complex and divergent movements that were a reflection of the crisis of the preceding model and the search for a new direction. The British context was a clear example and a precursor of many of the problems and transformations that were occurring. The United Kingdom especially suffered from the industrial offshoring process caused by globalisation and the impacts of that were strongly noted in its metropolitan regions, where the production resources were concentrated. This caused the idea of “urban decline”, understood as a pressing matter, to acquire a central position in the public debate (Cheshire and Hay 1989). The semi-abandoned physical landscape left behind by the flight of production resources served as a tangible reflection of the collapse of the industrial world and of the multiple social problems deriving from the increase in unemployment. It is within this context that the ideas that economic growth is a prior requirement for social welfare and that our cities are spaces where the battle for reconstruction will take place began to take root (Cochrane 2004).

The way to tackle this new outlook was neither immediate nor univocal. The confrontation between the neoliberal policies of Margaret Thatcher and the brief experience of municipal socialism (Boddy and Fudge 1984) shows how the discussion regarding the possible alternatives took place within the same national sphere. The contrast between these two stances also reveals agreements on two influential ideas that took hold at this time: the new relevance of the local scale and the new value of culture.

Both Thatcherism and municipal socialism defended the city as a space of strategic importance. The new right contemplated the urban areas as places where to make the rhetoric of the national economic regeneration visible, also filtering the idea of the convenience of privatisation and investment in large infrastructures for managing the transition (Barnekov et al. 1989). Paradoxically, this narrative was reconciled with a governance model leaning towards centralisation. On the other hand, for the new left that had managed to take over local governments of significance, the city was envisioned as a place of resistance against the central state project and as a trench from which to test grassroots policies of an innovative nature, susceptible to providing alternatives to the neoliberal advance and the old Keynesian labour movement simultaneously (Bianchini 1989). With hindsight, municipal socialism’s vision of the local scale unveils a clear idealised and defensive character. Combined with the movement from the right and fed by different discourses that underlined the importance of cities in the global scenario (Castells 1989; Sassen 1991), it contributed to further cementing the misleading and persistent myth of cities as autonomous entities and as places brimming with opportunities.

In the cultural field, municipal socialism identified a strategic space to boost the transformation that it argued for. The rise of advanced modes of cultural production and the associated labour market expansion gave birth to the idea of “cultural industries”, an economic sector of rising importance that was seen as a niche of opportunity for the reconstruction of the British production model without relinquishing its industrial tradition (Cochrane 1986). Beyond the macroeconomic perspective, the recognition of the economic dimension of culture had an added political aim, as it was understood as a front of action for improving the working conditions of the cultural agents, favouring labour inclusion by paying attention to the auxiliary jobs and shattering the elitism of which the paradigm of cultural democratisation was accused (Garnham 2005). In contrast, during the dismantling of the Welfare State carried out by neoliberalism, the usefulness and legitimacy of public investment in culture were cast into doubt. Because of this, cultural policy was obliged to justify its contribution on the basis of new demands, such as economic, social and urban development (Belfiore 2004; Subirats et al. 2015). In the right-wing political framework, the idea of cultural industries (production-based) was replaced by that of “economics of amenities” (consumption-based) (McNulty et al. 1985). Under the latter perspective, cultural assets were used to capture international attention, by attracting tourists and investment, and boosting property development. Once again, the diverging postures ended up giving rise to an unexpected convergence: culture became a space *from* which to act and not only *in* which to act.

### The practical preamble: Glasgow 90 (and Barcelona 92), the creative city acquires body

The creative city approach is not only constructed within the realm of theory. The exercises of policy design and its implementation act as additional vertices in a continuous process of cross-triangulation (Bianchini 2018). This is a constant and characteristic trait which is reflected in the abundance of conceptual reviews, discursive analyses and case studies in academic literature. In fact, the applied experience, guided by the comprehensions which we saw flourish in the previous section, had a crucial role as a preamble for the formulation of an initial policy proposal for the creative city.

The transition from the 1980 s to the 1990 s saw how a large number of European cities went through profound transformations characterised for including arguments associated with culture. The publication *Cultural policy and urban regeneration: The West European experience* (Bianchini and Parkinson 1993) becomes a bibliographical reference of great interest for the way in which, through the analysis of eight cities belonging to six different countries (United Kingdom, Netherlands, Spain, Italy, Germany and France) gives an account of the panorama that was unfolding, as well as the opportunities and conflicts that could be made out on its horizons (Bianchini 1993a).

Although the experiences compiled in the book were heterogeneous and reflected a path dependency that went beyond the local scale, their general willingness was aligned with the mindset introduced in the previous section: urban regeneration, economic reconstruction and improvement of the local-national image on the global stage (Bianchini 1993b). Therefore, the approximations prioritising economic growth by treating culture as an amenity pre-dominated, but it is important to note that these not only included a high range of degrees and declinations, they also coexisted with other forms of action. In particular, frequent cultural strategies and projects aimed at community development and/or caring for run-down urban areas (Belfiore 2002). These type of approaches reflected the demands that were being placed on cultural policies to demonstrate their public value and were also related to a certain persistence of the sociocultural animation and community arts projects within the advance of the cultural management paradigm (Kelly 1984).

Although clear frictions are observed between the approximation from the economic aspect with the global perspective and the approximation from the social aspect with local focus, these also show a certain complementarity (Pratt 2010). The eight cities presented in the book integrated both perspectives, the differences being in their orientation, connection and balance.

The possibility of combining objectives and diverse forms of action to achieve crosscutting change was illustrated with the experience that opened the practical section: the urban transformation of Glasgow based on its designation as *European City of Culture 1990* (Booth and Boyle 1993), a case which at the moment of the publication of the book was already recognised as a model for success. Glasgow was the perfect embodiment of a British city economically and socially broken by the deindustrialisation. Its regeneration was directed by the City Council, but, as is sometimes forgotten, it could not have been carried out without the decided support of the central government, which as we said needed clear images to disseminate - both internally and externally- the message of national reconstruction. Nor could it have been done without the support of the European Commission, which reformulated the ECoC programme that served as a vehicle for the process (García 2005; Immler and Sakkers 2014).

The regeneration of Glasgow deployed actions which in that period were already considered close to conventional: cultural flagships, regeneration of the riverfront, tourist marketing, moderate decentralisation of urban interventions, the search for community support, etc. However, the element which served to lend distinction to the experience and draft the success story was the commitment to the cultural industries -mainly art, design and audiovisual- as a resource for economic and urban reinvention. On that basis, and directly connecting with the experience of municipal socialism, the path was opened towards a new economy that presumed to restore the skills of the local working class labour force (Booth and Boyle 1993). Glasgow presented itself as a city that was rebuilding itself through culture, *creatively* converting its weaknesses into strengths. It is important to note that Glasgow’s transformation strategy was designed by Comedia, a consulting firm headed by Charles Landry, who admitted that the first time he had ever used the concept of “the creative city” was in the title of the document they prepared (Landry 2005). As we have mentioned, applied experience served as a test for intuitions, linking theoretical thought with a strong practical vocation.

To close this section, it is worth briefly mentioning Barcelona 92 as a somewhat similar experience but, at the same time, contrastive to that of Glasgow 90. Here we have another great urban transformation, within the same period and which employed most of the actions listed in the first lines of the above paragraph. However, between both examples there are differences that go beyond the nuances. The baseline objective of Barcelona 92 was not so much the urban relaunch through an overhaul of the economic model, but rather to reflect the re-establishment of democracy in Spain and the integration of an idea of modernity in accordance with the European canon (Molas 1991). That is to say, in the case of Barcelona 92, culture was employed from an erudite and civilising perspective rather than economic. The interventions in the public space and on the urban landscape became the cornerstones of its strategy (Borja 1995), understood as the means through which to develop civic pride and construct a sense of a collective project.

Although there are occasional mentions of Barcelona 92 in the book by Bianchini and Parkinson, the repertoire of case studies chooses Bilbao as a Spanish example, this being a transformation which stands apart from that of Barcelona for the greater centrality of the idea of urban-economic reconstruction in the post-industrial world. This detail is expressive of how the Anglo-Saxon viewpoint introduces biases in the construction of a comprehension of the role of culture in urban policies, emphasising certain aspects while discriminating others.

### The synthesis: an initial policy proposal for the creative city

As we have seen so far, different lines of analysis and the testing of a series of new trends progressively set out a complex understanding of the links between development, culture and city. In relation to this, a field of research that gained relevance was the one that sought to characterise the logics of production and consumption of the new capitalism. On the basis of the identification of an economic dynamic characterised by flexibility and for displaying complex geographic patterns (Sabel 1989), the call for attention towards the growing importance of aspects of a symbolic type in the global flows of exchange (Lash and Urry 1993) and the focus on the competitive advantage that changing from a labour-intensive model to a knowledge-intensive one meant (Porter 1989), “cultural economy” became an area of study that also acquired sophistication and acknowledgement (Pratt 1997; Scott 2000).

Having a solid analytical support, at a time when public policies aimed to work through evidence-based technical criteria in order to reaffirm their legitimacy (Young et al. 2002), acted as a driving force behind the programmes that promoted the development of the cultural and creative sectors for an economic turnaround. Furthermore, the influence of the studies of cultural economy was even broader due to the way in which they prepared the way forward for discourses that pointed towards the importance of culture, creativity and innovation within the entirety of the urban governance (Scott 2014). Although this new focus on urban creativity was conceptualised in different parts of Europe (particularly in Germany by the cultural policy consultancy STADTart, who established eventual collaborations with Comedia), the UK context continues to play a central role in its development and circulation.

At this point, the contact between Peter Hall, Franco Bianchini and Charles Landry acquires particular importance. The exchange of ideas between them set the tone for the first presentation of the creative city as an articulated policy proposal. Hall had dedicated years to working around the idea that, throughout history, the cities that have had the greatest moments of splendour had done so thanks to having configured themselves “creatively”. The energies to do so were born of the concentration of people, ideas and skills to which they are home, elements that establish a “creative milieu” (Hall 1998). As Hall indicates, creativity was not only a key for success, but rather a natural tendency of the city that was necessary to understand and stimulate.

For his part, Franco Bianchini introduced the notion of cultural planning, a concept that had arisen in the US and Australia but which he addressed from a European point of view, granting centrality to the idea of “cultural resources” (Bianchini 1999, 2016). The key here was in the way in which these resources were defined. Bianchini’s proposal goes beyond the conventional idea of material assets (works of art, built heritage, museums, cultural centres, etc.) to include elements of a more complex nature such as memories, citizens’ identities, shared values, lifestyles, democratic sturdiness, the propensity to civic engagement or the storytelling that revolves around a city. Bianchini also indicated that these resources affected matters that went beyond the understanding of culture as a sector, owning potentialities for a crosscutting action. Understanding how to activate and mobilise them was the basis of an endogenous development model that went beyond the scope of urban regeneration. From the perspective of cultural planning, culture was no longer a mere instrument to restore cities in decline, but rather a complex dimension that concerns the entire urban dynamic and public life.

The combination of these ideas, supported by the practical experience of the authors, served to present a preliminary proposal of *The creative city* (Landry and Bianchini 1995) that warned of an “urban crisis”, recognised a “time of transition” and called for a more holistic thinking and greater risk acceptance when responding to the cities’ challenges. In order to discover unforeseen opportunities, the creative approach needed to challenge the overestimation of the role of instrumental rationality in policymaking. It was also indicated that urban governance and city development should focus on the smart use of local resources rather than globalised formulas. As the authors state (Landry 2012; Bianchini 2018), this vision was a reaction against the generic, technocratic, top-down and cataclysmic urban transformation model which was extending throughout the West.

This creative city in its germinal stage was presented in a book of little more than fifty pages. Half of them formulated a conceptual framework which acknowledged Patrick Geddes, Lewis Mumford and Jane Jacobs as theoretical references. The other half of the work consisted of the listing of multiple examples, many of which were trivial and little known, that sought to transmit the idea that urban creativity, in the terms on which it was considered, was not a question of epic deeds, but rather something that happened repetitively, unperceived but successfully.

A few years later, Charles Landry was responsible for taking that rough draft of the creative city and converting it into a more detailed and acceptably cohesive policy proposal. His book *The creative city: A toolkit for urban innovators* (Landry 2000) once again called attention to the crucial importance of “rediscovering urban creativity” and, from there, built an articulated framework combining the perspective of cultural planning with that of urban governance. Landry’s exposition was punctuated with a large number of strong ideas, showing a special ability to formulate attractive statements acquired in the field of consultancy. This does not mean to say it is an exercise full of hot air. His central thesis, the idea that gives unity and meaning to the explanation, is that urban creativity is not defined in terms of production or consumption (Cunningham 2012), but rather in terms of process. In the words of Andy C. Pratt, Landry’s proposal “is about an inclusive and participatory city where arts and culture are a means and a practice of place making and living” (Pratt 2008). Additionally, it is appropriate to note that the choice of the term “toolkit” in the subtitle of the book has frequently been used by the detractors of the creative city to reduce it to an aspirational and formulaic proposal that leads to naive solutions. It is a criticism that does not correspond to the content of the publication, which places the weight of its attention on defining conceptual and methodological premises rather than giving specific instructions to follow. One of Landry’s declared objectives is to influence policymaking and urban governance. His discourse is formulated and presented from this point of view and this is how the emphasis in the applied side of the creative city and the use of the examples that illustrate the explanation must be understood.

Our assessment of Landry´s work is far from uncritical, which on the other hand reflects many of the preconceptions and contradictions of its epoch in the form of disproportionate optimism; focusing on the opportunities rather than on the problems; a vague hierarchy between heterogeneous objectives; the assumption of a corporate language that insists on economic reasoning; discrediting the bureaucratic logic in favour of ambiguous flexibility; a harmonic and virtuous idea of citizenry; entrepreneurial rhetoric surrounding the creative sectors or a misrepresentation of the city as a stand-alone entity. It is easy to recognise the origin of many of these inclinations in the historical itinerary that we have described. Taking into account the specific moment in which Landry’s book was published, it is essential to also consider the climate of enthusiasm (lately tempered) generated in the United Kingdom by the victory of Tony Blair in 1997, with a government project that announced a new importance of culture and, specifically, creativity (Banks and O’Connor 2017). In fact, Franco Bianchini identifies a slight shift of tone between the creative city discourse he and Landry presented in the 1990 s and the one that was proposed in the year 2000. In his opinion, that shift could be defined by a move from a radical and participatory willingness towards a more self-referential and technocratic elite style (Bianchini 2018).

Although Landry’s policy proposal had a big success, a couple of years later Richard Florida burst onto the scene with the “creative class” theory (Florida 2002) and rapidly got all the attention in the debates that linked creativity and city (Peck 2005). Even if both proposals (the one formulated by Landry and the one by Florida) tend to be cited together as if they were analogous approaches, the differences between them are more than significant. Florida’s proposal draws from diverse sources and uses them in a very particular manner. Jane Jacobs acts as a shared reference with Landry due to her vision of the good city as a socially vibrant space that functions as a generator of well-being (Jacobs 1961). Particularly in Florida’s discourse, but also in Landry’s, clear influences can be found of the descriptions that drew attention to the remarkable changes in contemporary urban lifestyles and the rising importance of leisure and cultural consumption (Zukin 1998; Brooks 2000). Even so, the theory of the creative class fits better in the field of studies of the new economy, its dynamics and its geographies. In the manner in which it is conceptualised, the creative class is an asset that offers a competitive advantage in the face of the reduction of production costs. The claims presented by Richard Florida that go further than this, as the representativeness of the preferences assigned to this specific group of people or their supposed propensity towards civic engagement, usually become the centre of attention when they are actually the weakest and most secondary elements of the discourse (Glaeser 2005). Despite substantial discrepancies, Richard Florida’s perspective is closer to that of Alan J. Scott when working around the geographic logic of cultural production ecosystems (Scott 2000), or that of Ann Markusen when she places artists in the centre of the analysis and proposes assessing their contribution to the regional economies by defining an “artistic dividend” (Markusen et al. 2004; Markusen and Schrock 2006). Our intention is not to establish a valuation hierarchy between one type of approximation and the other, but rather to introduce a certain order between ideas that have tended to become mixed much too lightly.

Lastly, it is worth discussing another extended idea, the one that argues that the theory of the creative class had an overwhelming impact on a global scale. It is undeniable that Florida’s discourse became a circulating policy model that made its way around various territories (Peck 2009), but this does not mean that its influence had a homogeneous reach. This affirmation is particularly reflected in the European context, where the dynamics described by Florida from a US perspective do not correspond very well in functional terms (Martin-Brelot et al. 2010) and also cause frictions with the region’s common values.

It is on this last point that we will close our review of the emergence and consolidation of the creative city discourse and its transformation into an -open but specific- approach. We will show that, in the international arena and particularly in Europe, the policy proposal distilled by Charles Landry has had a considerable influence, although its acceptance has been subject to a process of correction and adjustment in order to create a comprehension of the creative city in accordance with more consensual frameworks.

### Adaptation and acceptance: the creative city under the prism of sustainability

We have explained that one of the factors that surrounded the emergence of the creative city discourse was a shift in the understanding of the value of culture, mostly motivated by an increasing attention to its economic dimension. As globalisation advanced, culture became an object of intense debates, but the above was not the only way towards which the arguments leaned. The need to delimit the treatment of cultural products and services in the global market intensified the defence of the intrinsic value of culture, which in the scope of international organisations was reaffirmed by concepts such as “diversity” (UNESCO 2001) and “cultural rights” (The Fribourgh Group 2007). The emphasis on the inherent importance of culture also gained recognition within the framework of sustainable development with the assimilation of the idea of the “fourth pillar”(Hawkes 2001). The reformulation of the concept of development formulated by Amartya Sen (Sen 1999), understood as a process that expands the capabilities to live a good life, connected with this entire movement endorsed internationally (PNUD 2004). Recent contributions such as the *2030 Agenda for Sustainable Development* (ONU 2015) reflect the integration of culture within the prisms of sustainability and human rights.

The idea of culture for sustainability was constructed at the same time as the creative city vision and, in the same manner, needed a process of gradual structuration. It would be a mistake to assume that this process only involved large international organisations. To the contrary, the agents that took part in it -creating narratives and applying pressure, contributing to the progress proactively or approaching it in an opportunistic manner- are numerous, varied in nature and closely connected to the territory; these include civic associations, cultural agents, regional and local governments, coordinating platforms, transnational networks, and so on (Pascual 2021).

It would also be wrong to consider the culture for sustainability perspective as a complete alternative to the culture for urban development and economic growth focus, as a paradigm arising within a totally different context of forces and trends. To such an extent did the culture for sustainability perspective participate in the reality described in the previous sections that, the Universal Forum of Cultures celebrated in Barcelona in 2004, a top-down urban transformation project based on the mega-events formula, served to stage its consolidation (Majoor 2011).

The culture for sustainability perspective does not turn its back on the studies of the economy of culture, as it draws multiple statements from them. This is particularly perceptible within the context of the European Union, which has logically assimilated discourses that arose, to a large extent, within its geographic scope. For example, the *New European Agenda for Culture* (European Commission 2018) assigns a key importance to creativity to reinforce the economic dimension of culture and calls for it to be connected with education and innovation to foster the creation of jobs and growth. Even so, the objectives of an economic nature are placed second, behind the social goals, which are framed within a discourse clearly influenced by Sen’s idea of development, highlighting welfare and quality of life as emerging fields for cultural action and introducing concepts such as “cultural capacity”. The latter acquires particular interest, as it calls for "making available a wide range of quality cultural activities, promoting opportunities for all to take part and to create, and strengthening links between culture and education, social affairs, urban policy, research and innovation” (European Commission, 2018 , p. 3). It is possible to affirm that the idea of culture as an element that can take part in other public policy areas in favour of comprehensive development is akin to the vision of creativity that Landry and Bianchini proposed in the mid-1990 s.

Additionally, the European Union has shown a growing interest in promoting cities, seeing them as potential nodes for the economic and cultural cohesion of the region (Eurocities 2017). The urban policy programmes promoted by the European Commission from this angle also reflects the specific influence of the creative city policy proposal (Vinci 2008). The *Cultural and Creative Cities Monitor*^1^ is one of the more obvious examples, to which the experience of the *Urban Innovative Actions*^2^ can be added. The latter presents a strategic framework structured into different topics, with one dedicated to “culture and cultural heritage” that includes objectives in relation to social cohesion, improving regional competitiveness, innovation for governance and contributing to “culture-centred participatory urban processes”. Once more, the holistic and crosscutting overview which Landry and Bianchini’s proposal claimed for urban governance is reflected through the idea of creativity.

In any event, it is important to highlight once again that the integration of the creative city perspective within the framework of culture for sustainability does not result in a refined paradigm or a general model for action. The study of cases in different geographic realities continues to account for the wide range of intervention modalities and the importance of the contextual factors regarding applied experience (Culture for Cities and Regions 2015). As Franco Bianchini indicated very early on (Bianchini 1993b), the implications of path dependence include the national attitudes towards culture, the local urban planning traditions, the inertias of public policies, the distribution of power within the systems of government, the relationships of force between market dynamics and social movements, and the permeability to external influences. Furthermore, the importance of context -which includes time and place- is not to be understood mechanically, but rather in terms of complexity, it being necessary to assume that the policies that explore the intersections between culture and city act in fields of unstable chemistry (Comunian 2011).

In summary, the creative city policy proposal has managed to connect with the sustainable development perspective and, from there, it has transitioned towards international urban and cultural agendas. By doing so, the creative city has positioned itself in a policymaking framework which nowadays has a high degree of consensus on a global scale and especially within Europe. The creative city discourse has introduced diverse aims and almost literal statements within the culture for sustainability perspective. In the other direction, by coming under the prism of sustainability, the creative city came into contact with concerns and ideas which, even though they are familiar to it, provide a new mould for its conceptual and operational apparatus. The sustainability perspective reinforces the arguments of the creative city which called for an understanding of the cultural dimension of the urban environments as a complex ecosystem, which exceeds both the sectorial understanding of culture as well as its instrumental usage. In any event, the creative city framework is not completely emulsified into the discourse on sustainability, which is also configured as an assemblage of ideas rather than as a closed equation. In this regard, the calls for creativity in current urban policies continue to reflect the origins and the route followed by the creative city through its emergence and consolidation, but now they are fitted into a larger and more complex framework, the principal function of which is to provide guidance. This is why, at this point, it is especially appropriate to understand the creative city as an “approach”; as an epistemological and methodological focus that, as mentioned, was originally conceived to manage periods of transition.

## The prospects of the creative city in a new time of transition

Although we have focused on its theoretical and discursive component, we have also indicated that the creative city approach is constantly nourished by practical experience. The complex overlap between vision and execution (Doyle and Mickov 2019), emphasised by the abundance of strategic orientations and modes of action, complicates the task of presenting a general assessment of the contributions of the creative city. This point, which in fact generates noticeable discrepancies, is central when discussing the validity and limitations of the approach.

While the debate is frequently reduced to a black or white confrontation between those defending the creative city as a virtuous paradigm and those that present it as a mere lubricant for the neoliberal city, the descriptions that speak of entangled interventions and partial materialisations are more nuanced and plausible. From this perspective, the creative city approach is often obliged to coexist with other urban and cultural policy models, and is frequently used as a rhetoric device for conventional economic development strategies (Grodach 2013). This leads us to think of an approach that is less hegemonic than what it sometimes appears to be, affected by instrumentalisation when not, simply and plainly, by confusion. The lack of understanding of its theoretical premises, its ambitions and its dilemmas is, to a large extent, the reason why the creative city becomes a hollow concept or a repertoire of clichés when facing action. We think that it is worthwhile to resituate the creative city approach by deepening its understanding. Our exercise to review its construction process aims to be useful in this regard.

However, our explanation underlined the fact that the creative city was defined in a specific period and place. In spite of its evolution over time, the creative city approach conserves certain preferences and biases rooted in its origins. The extensive academic literature on the creative city has pointed out all its flaws, of particular significance being the pre-domination of economic objectives even when coexisting with other goals (Evans 2017); prioritisation of growth losing sight of redistribution and equality (Gerhard et al. 2017); opportunistic attitudes deriving from the insistence on finding opportunities (Chatterton 2010); poor use of the possibilities of culture, sometimes reduced to a mere question of image and storytelling (Vanolo 2008); promotion of a cosmopolitan culture over local or indigenous styles (Pratt 2011); idealised and non-representative characterisation of cultural and creative agents (Markusen 2006); primacy of an entrepreneurial orientation that marginalises the majority of the cultural community (Ponzini and Rossi 2010); the way in which a discourse formulated by the urban elites contributes to the reproduction of disadvantages and inequalities (Leslie and Catungal 2012); and the tensions and confrontations that sometimes arise from these exclusions (Novy and Colomb 2013; D’Ovidio and Cossu 2017).

If in these matters, and practically right from the first moment (Bianchini 1993a), the feebleness of the creative city have been foreseen, the persistent crisis which began in 2008 and the need to find new points of reference will accentuate the tensions surrounding the approach (Harris and Moreno 2012).

Today we find ourselves in the midst of a transition scenario of a problematic nature, defined by the increase in inequality, the normalisation of precariousness (affecting the economic and political structures), and the growing tensions between central spaces and peripheral realities (from a social and geographical point of view). The misgivings and discomforts that this situation produces are directly reflected in the cultural field, emphasising its conflictive dimension rather than its condition as an arena for mutual recognition and unity. Vigorous progressive movements, in favour of a new generation of politics of representation, coexist with reactionary trends that contribute to the advancement of new nationalisms that defend an idea of culture which is historicist, hermetic, static and anchored in traditional values. This second dynamic clashes against the growing diversity of the urban populations, which finds an additional challenge in the inclusion of migrants and refugees. The contemporary cultural wars are based on disinformation and on relativism, incrementing the ideological polarisation and impeding public debate. In the area of government, all of these tensions feed statist postures, centred on security and based on authority. The leadership capacity that was presumed to local governments become highly dubious in the light of the threat of seeing their resources and powers once again reduced. Seen from a broader perspective, the relation of the problems listed with an unsustainable global development model becomes tangible, with the climate crisis being one of its more pressing, alarming and unpredictable consequences.

The description of such a turbulent panorama will give new arguments to those who judge that the creative city has become an outdated proposal with no traction capacity (O’Connor and Shaw 2014). This statement, however, seems slightly hasty if considering the significant influence that the creative city still exerts on the urban and cultural agendas; if performing the task of critically reviewing its conceptual basis and its various evolutions; and if paying attention to a number of positive trends emerging during these convulsive times to which the creative city, understood as an approach, could contribute while discovering new concerns and ideas to reconfigure its outlines.

The last part of this section will be devoted to completing the argumentation we have constructed, pointing towards six emergent trends which are attempting to consolidate themselves and with which the creative city approach could establish mutually reinforcing synergic relationships:

### Rights and justice for a new comprehension of the city

Urban realities are spaces in which many of the challenges of the contemporary world manifest themselves. In contrast to the “millennial tone” that pervaded the creative city discourse in the 2000 s, urban challenges are currently understood in terms of problems rather than of opportunities (Florida 2017). The city is no longer considered as a mere element for production or consumption. Instead, a call is made to envision urban environments as contexts for personal and collective development and as places that must guarantee the coverage and exercise of fundamental human rights (Fainstein 2014; Grigolo 2019).

Another notable change is the deterioration of the idea that cities have creative agency and engender innovation spontaneously (Van Damme et al. 2018), which was central -and still is- in the creative city discourses. Questioning whether creativity is a natural propensity of cities leads to a greater politicisation of the creative city and this can incorporate the recovery of the idea of “the right to the city”, which is nowadays enunciated in a strongly activist and radical way (Harvey 2012; Novy and Colomb 2013).

Under this view, cultural rights demand greater recognition as an integral part of the right to the city and the *Agenda 21 for culture* (UCLG 2004), that was presented when the creative city enjoyed great popularity but never really connected with it, becomes an important reference to pay attention to the cultural dimension of urban policies.

### Well-being for a broader understanding of the value of culture

The creative city emerged at a time when increasing attention was being paid to the capacity of culture to generate externalities. The use of culture for urban development and economic growth (Vivant 2007), an idea towards which part of the statements of the creative city pointed to, was only one of the fields of action that were explored. Published by Comedia, the book *Use or ornament?* by François Matarasso drew attention to many other opportunities, including intergenerational contact, skill-building and employment, or community empowerment (Matarasso 1997). Matarasso’s discourse had a notable impact on the debates on culture and was specifically combined with the insights of Charles Landry and Franco Bianchini on urban regeneration (Landry et al. 1995).

Although discussions around the instrumentalisation of culture tend to emphasise its most problematic aspects, it can be argued that it helped to forge a wider understanding of the value of culture (Gibson 2008). In fact, scientific analyses are less dichotomous nowadays in their understanding of what the proper and improper objectives of cultural action are, firmly recognising the importance of culture in matters such as health or psychological well-being (Grossi et al. 2012).

Studies that address the role of culture in local development have incorporated this enriched comprehension, overcoming narrow visions and mono-causal understandings (Sacco et al. 2014). Therefore, in this increasingly complex understanding of the value of culture another opportunity is found to revise and reinforce the creative city approach.

### Crosscutting cultural policies that call for a new centrality:

The growing attention to the capacity of culture to contribute to human development and generate social value brings multiple fields of research and praxis into the light. In many cases, these accumulate a long-standing experience, with education (Grupo de Educación de Matadero Madrid 2017), health (Gordon-Nesbitt 2015), inclusion (Baltà Portolés 2016), intercultural dialogue (European Agenda for Culture 2014), the enhancement of public space (Toolis 2017) and environmental commitment (Arts Council England 2020) being some of the topics in which the arts and creativity claim importance. These new spaces for cultural action (Segovia et al. 2015) are located beyond the classic sectorial boundaries and also break with the conventional strategies for economic development, tourism or attractiveness. They exemplify and work towards a possible new approach to cultural policies, which could serve to resolve the exhaustion of the models of democratisation and cultural management.

If we draw attention to this special dynamism in the field of cultural policies it is because it could be helpful in solving one of the main pending issues of the creative city approach. Paradoxically, creative city strategies often have little interaction with cultural policy departments, with areas such as urban planning, economic development or tourism promotion being more frequently involved (Grodach 2013). There is now a window of opportunity to link the emergence of innovative cultural policies with the creative city approach. This relationship is particularly coherent as both share a cross-sectoral and experimental outlook.

### The search for social innovations in the urban context

The spaces neglected by the withdrawal of public action due to the crisis of 2008 saw innovative processes flourish, many of them driven by civic initiatives (Walliser 2013). These were not only interesting due to the way in which they achieved to respond effectively to unattended needs, they also managed to generate transformative social practices and construct new shared values (Subirats and García-Bernardos 2015).

In their search for new formulas for intervention and development, public policies in general and urban policies in particular have paid considerable attention to this type of initiatives, trying to promote them, institutionalise them and scale them up. This manner of observing innovation overcomes its technology-centered comprehension and emphasises its social and political nature. Even so, it is possible to contend that approaches to social and urban innovation continue to be made from a primarily scientific focus; the constant emphasis on the need to create “solutions” serves as an example.

The creative city approach, particularly in its most radical early formulations, is closely related to that of urban innovation. The connection between the two is clearly exemplified in “creative placemaking” (Markusen and Gadwa 2014) and “urban manufacturing” (Savini and Dembski 2016), two emerging frameworks that Carl Grodach defines as “new creative city movements” (Grodach 2017). The transformative capacity of cultural action, the way in which creativity incites experimentation or the disruptive potential of the arts are some of the valuable issues that the creative city approach introduces in the field of urban innovation. As Bianchini and Landry pointed out when reflecting on this issue (Landry and Bianchini 1995), creativity is a divergent process while innovation has a convergent logic. Both need one another.

### Redefinition of the concept of governance

In it is early formulations, linked to the notion of New Public Management (Schedler and Proeller 2005), the idea of governance was proposed in favour of seeking efficiency. To do so, it was advocated to introduce complementary and corrective capacities for public action in the form of expertise, independence and monitoring. The emergence of a civic vision that demanded the possibility of a broader, more plural and more direct political participation (Harvey 2012) reconfigures the concept of governance by converting it into a matter that is defined in terms of deepening democracy rather than technical improvement (Melo and Baiocchi 2006). This trend, which connects urban and cultural debates (Baltà Portolés et al. 2014), could also contribute to correct the shift that Franco Bianchini identifies in the evolution of the creative city discourse, from a participatory willingness to a more technocratic style (Bianchini 2018).

It is also interesting to point out that, in contrast with the anti-planning mentality that spread in the 1980 s and influenced the early formulations of the creative city, the political transformation that is sought tries to connect with the logic of the public action rather than break ties with it. Charles Landry’s recent defence of bureaucracy (Landry and Caust 2017) marks an enormous novelty in relation to a subject that, in the initial policy proposal of the creative city, was treated only in terms of disaffection. That this novelty does not appear incoherent reflects the capacity of the creative city to incorporate new inputs and reconfigure itself.

Lastly, as regards to the new understanding of the city that we have described above, addressing urban problems from a closer perspective reveals the limitations of local governments while debunking the myth that cities had become key players in the contemporary world who could reorient themselves with high levels of autonomy thanks to creativity. In the light of this, a reformulation of the multi-level governance structures is demanded (Blanco and Subirats 2012), with cities still exerting a strategic role but in more differentiated ways (Le Galès 2018). This adds complexity to the creative city approach concerning its call for urban governance.

### Planning for uncertainty

The growing instability around the current world is leading to more complex understandings of reality. As we have indicated, this affirmation is especially suitable when referring to the interrelations between culture and cities. Experimentation gains importance and, with it, the aim of planning is no longer to control its context of action but rather to understand its intricate configuration in order to be able to generate progress in a desired direction.

New discourses on urban governance and city development ask that importance be assigned to the learning acquired throughout the process and not only on the objectives to be achieved, that the ideas of success and failure be refined and that tactical attitude be combined with a strategic perspective (Sendra and Sennett 2020; Marrades et al. 2021). With these arguments, some of the statements contained in the first draft of the creative city are once again repeated (Landry and Bianchini 1995).

The comeback of these ideas is particularly important, as it relativises one of the most successful areas of creative city studies, which would be the one that tries to measure all its attributes and anticipate the results of any intervention (Campbell et al. 2017). Without denying the importance of analysis and evaluation, in this unstable epoch it seems necessary to overcome the limitations of a certain logic of instrumental rationality and move away from our culture of risk aversion. The creative city approach can be inspiring for such a purpose.

**Fig. 1:**
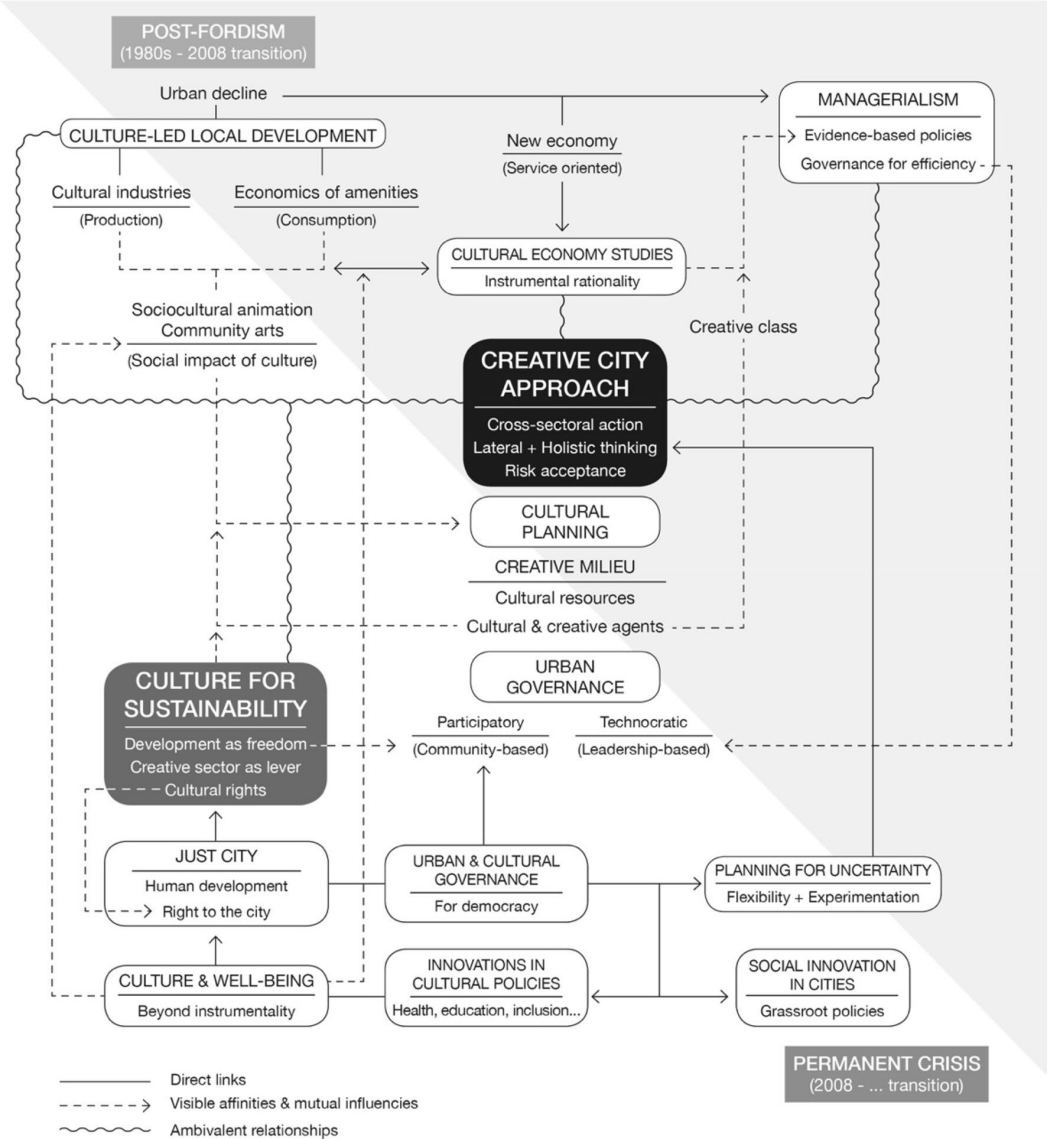
The creative city approach, initial construction and possible reorientation

Finally, the following diagram represents a synthesis of the preceding argumentation, while portraying the relations between the main elements that influenced the initial construction of the creative city approach and those new trends that could be useful for its fruitful reformulation (Fig. 1).

## Conclusion

We have dedicated the first part of this article to review the process through which the creative city was assembled as a reasonably articulated and transnationally validated framework. Although at first sight it may seem slightly contradictory, we have sought to reflect the multiple and divergent interpretations that surrounded this process and played a role in it, while at the same time indicating the existence of a distinguishable epistemological and methodological focus. Thus, the creative city is presented as an approach, as a distinctive and at the same time malleable framework. This malleability is valuable when placing the creative city approach under new concerns that contribute to its reinvigoration; the manner in which the creative city approach was integrated into the prism of sustainability serves as an example. As a counterpoint, the malleability of the creative city approach reveals limitations due to specific biases, linked to its origins, which are deeply rooted in its conceptualisation. The possibility of resolving these constrictions in order to provide a renewed usefulness to the creative city approach requires an adequate understanding of its motivations and dilemmas.

The understanding of the creative city as an approach appears to be pertinent for two main reasons. The first is the capacity that this policy proposal retains to influence within the fields of urban and cultural policies. Paradoxically, in spite of this, the creative city functions to a large extent as an empty signifier that depends on who gets to assign meaning to it. Therefore, that its political potential is used positively is a matter in contest. Secondly, it is necessary to recognise that the creative city approach contributed to introduce a complex comprehension of the cultural dimension that urban environments, public life and the governance of the city possess. Such an understanding is equally far from being fully assimilated, even at a time when the main challenges of urban societies have a marked cultural component.

It is on this last note that the reflection we have proposed achieves its main relevance. We find ourselves immersed in an epoch of problematic transition, in which culture and the city are alternatively defined as spaces of conflict or spaces of hope. The prevailing unrest adds pressure on the traditional weaknesses of the creative city, which could definitely be seen as a docile proposal with no transformative capacity. A less pessimistic view recalls that the creative city was formulated originally as an approach to cope with times of transition and, in addition, identifies an incipient set of changes of understandings and ways of action that could find support in the creative city approach and, in turn, positively contribute to its reformulation.

The revitalisation of the creative city to advance towards a new horizon or its progressive cornering until its disappearance are two plausible possibilities which will be determined with the passage of time. In one way or another, the review of the emergence and transnational validation of the creative city approach shows how new visions are forged within contexts of transition. On the other hand, the claim that the potential value of the creative city wants to draw attention to the fact that these new insights do not just appear out of thin air, but rather they are politically built, using the preceding foundations and the cracks that let us see the future. Today, the creative city approach is a useful resource in this sense, to which others should be added.

## Data Availability

Not applicable.

## Footnotes

1 https://composite-indicators.jrc.ec.europa.eu/cultural-creative-cities-monitor.

2 https://uia-initiative.eu/.
